# The pharmacodynamics of polymyxin B in *Acinetobacter baumannii* in murine thigh and lung infection models

**DOI:** 10.1093/jac/dkag097

**Published:** 2026-03-17

**Authors:** Sanne van den Berg, Michel Pieren, Sebastiaan D T Sassen, Willem A M de Jong, Catharina S C Boonman, Glenn E Dale, Anouk E Muller

**Affiliations:** Department of Medical Microbiology and Infectious Diseases, Erasmus MC University Medical Center Rotterdam, Rotterdam, The Netherlands; CATOR, Center for Antimicrobial Treatment Optimization Rotterdam, Rotterdam, The Netherlands; BioVersys AG, Basel, Switzerland; CATOR, Center for Antimicrobial Treatment Optimization Rotterdam, Rotterdam, The Netherlands; Department of Hospital Pharmacy, Erasmus MC University Medical Center Rotterdam, Rotterdam, The Netherlands; Department of Medical Microbiology and Infectious Diseases, Erasmus MC University Medical Center Rotterdam, Rotterdam, The Netherlands; Department of Medical Microbiology and Infectious Diseases, Erasmus MC University Medical Center Rotterdam, Rotterdam, The Netherlands; BioVersys AG, Basel, Switzerland; Department of Medical Microbiology and Infectious Diseases, Erasmus MC University Medical Center Rotterdam, Rotterdam, The Netherlands; CATOR, Center for Antimicrobial Treatment Optimization Rotterdam, Rotterdam, The Netherlands; Department of Medical Microbiology, Haaglanden Medisch Centrum, The Hague, The Netherlands

## Abstract

**Objectives:**

Polymyxin B has been used for many years to treat *Acinetobacter baumannii*, but little is known about its pharmacodynamics (PD). We aimed to describe the PD of polymyxin B in treating *A. baumannii* infections.

**Methods:**

Using the murine neutropenic thigh and lung infection models we determined the magnitude of the pharmacokinetic (PK)/PD index correlating with efficacy for eight *A. baumannii* strains. PD was analysed using the *E*_max_ model to determine PD targets. Using published human PK data the PTAs were calculated.

**Results:**

In the thigh infection model, stasis, 1-log_10_ and 2-log_10_ kill were reached for all strains. Median (range) *f*AUC/MIC (area under the unbound concentration–time curve divided by the MIC) targets for stasis, 1-log_10_ kill and 2-log_10_ kill were 2.1 (1.0–11), 2.9 (1.0–15) and 4.0 (1.1–20), respectively. In contrast, in the lung model, 2-log_10_ kill was reached in 2/8 strains only, and there was insufficient killing at tolerated exposures to determine PD *f*AUC/MIC targets. For the standard human dosing regimen, PTAs derived from the thigh model were inadequate at the USCAST (United States Committee on Antimicrobial Susceptibility Testing) clinical breakpoint and a protein binding of 90%.

**Conclusions:**

Whereas polymyxin B treatment had good efficacy in the murine thigh infection model, in the lung infection model its effect was limited. PD targets, with MICs of circulating *A. baumannii* isolates of ≤2 mg/L, are not reached with a standard human dosing regimen and will probably exceed threshold values for toxicity. These data suggest that the efficacy of polymyxin B as monotherapy for *A. baumannii* infections is questionable.

## Introduction

*Acinetobacter baumannii*, especially carbapenem-resistant isolates (CRAB), is an emerging cause of healthcare-associated infections. This is a significant threat to public health. The development of new antibiotics, repurposing of drugs approved for other pathogens, and dose optimization of disused antibiotics are crucial for treating CRAB infections in the future.

The polymyxins are an old class of antibiotics used to treat Gram-negative bacterial infections, including those caused by *A. baumannii*. This class includes polymyxin B, a compound that is administered IV in its active form. As polymyxin B was discovered in the 1940s,^1^ it was introduced to the market without solid pharmacokinetic (PK) and pharmacodynamic (PD) knowledge. Despite the urgent need for antibiotics active against Gram-negative pathogens, the use of polymyxin B was limited due to toxicity issues, mainly nephrotoxicity and neurotoxicity.^2^ Information on PK and PD is crucial in navigating the delicate balance between efficacy and toxicity.

Limited data are available on the PD of polymyxin B. Previous studies in murine infection models indicated that the PK/PD index best correlated with antibacterial efficacy is the ‘area under the unbound concentration–time curve divided by the MIC’ (*f*AUC/MIC).^3,4^ However, these studies were performed using *Klebsiella pneumoniae* and *Escherichia coli* strains. The magnitude of the PK/PD index correlated with a certain antibacterial effect was quite variable in these studies. For *K. pneumoniae*, the magnitude of the *f*AUC/MIC for a static effect was found to be 1.2–60.6,^3,4^ whereas for *E. coli* the static *f*AUC/MIC range was 27.5–102.6 in the thigh infection model.^4^ In the study of Landersdorfer *et al*.,^3^ a 1-log_10_ kill effect was reached in the three tested *K. pneumoniae* strains; this was reached in only one of four strains of the study of Van der Meijden *et al.*^4^ In both studies a 2-log_10_ kill effect was not achieved. This indicates that the antibacterial effect of polymyxin B may differ between species, and the overall effect may be limited. Especially in lung infections, the effect may be reduced by a structure-interaction between the polymyxins and lung surfactant or binding to mucin.^5,6^ Knowledge of the (magnitude of the) PK/PD index for *A. baumannii* is therefore required to optimize human dosing regimens used to treat *A. baumannii* infections. In the current study, the murine plasma PK of polymyxin B, as well as the subsequent plasma exposures, were determined in *A. baumannii*-infected mice. These exposures were subsequently correlated to the antibacterial effect against eight different *A. baumannii* strains to determine the PK/PD index and its magnitude required for various levels of killing in two murine infection models.

## Materials and methods

### Bacteria, media and antibiotics

Eight *A. baumannii* strains, all with polymyxin B MICs of 0.5 mg/L, were used for these studies. Except for BV566, all other strains were carbapenem resistant (CR). Isolates were grown, subcultured and quantified using CAMHB II (BioTrading Benelux B.V., Mijdrecht, The Netherlands) and Mueller–Hinton II agar (MHA; BD Benelux N.V., Erembodegem, Belgie). Polymyxin B sulphate (lot A1421526, Xellia Pharmaceuticals, Copenhagen, Denmark), consisting of 71% B1 and 22% B2, was dissolved in 0.9% physiological saline solution (Baxter Healthcare SA, Zurich, Switzerland). Solutions were freshly prepared on the day of the experiment.

### Neutropenic murine thigh and lung infection models

All studies in experimental animals were carried out in accordance with the EU Animal Directive 2010/63/EU 2010 directive^7^ as described before,^4^ with approval of the institutional Animal Welfare Body (IRN 2022-0004). Female outbred CD-1 mice, specified pathogen free, obtained from Charles River Germany, mean ± SD weight of 24.8 ± 1.7 g, 7–8 weeks old, were used. Animals were socially housed under standard conditions with drink and feed supplied ad libitum, and were randomly assigned to the experimental groups by an analyst who was blinded to the study details. There was no control for potential confounders and researchers were not blinded.

Neutropenia was induced by intraperitoneal cyclophosphamide injections at 4 days (150 mg/kg) and 1 day (100 mg/kg) before infection. For infection, an overnight *A. baumannii* culture was diluted in fresh CAMHB, incubated for 1 h at 37°C, and further diluted in CAMHB (for thigh infection) or saline (for lung infection) to obtain an inoculum of ∼4 × 10^6^ and 2 × 10^8^ cfu/mL, for thigh and lung infection, respectively. For thigh infection, mice were infected intramuscularly in each thigh (0.05 mL). For lung infection, isoflurane anaesthetized mice were infected intranasally (0.05 mL). Infections were induced at *t* = −2 h, corresponding to 2 h before treatment initiation. Buprenorphine (subcutaneously, every 12 h starting at infection) was applied as analgesia. The health status of mice (including weight, temperature, colour of eyes, ruffled fur, hunched back) was checked at least twice daily.

### Polymyxin B pharmacokinetics

Two hours after thigh infection with *A. baumannii* BV378, polymyxin B was subcutaneously administered (0.1 mL) in a range between 2 and 64 mg/kg, with two mice per group. Group size was based on results of previous studies.^3,4^ At 12 different timepoints, blood was collected in K3E EDTA tubes (Sarstedt, Nümbrecht, Germany) under isoflurane anaesthesia through orbital sinus bleeding, which was immediately followed by cervical dislocation. Samples were placed on ice. Immediately after blood collection, bronchoalveolar lavage (BAL) was performed. The trachea was exposed by a ventral vertical incision in the neck and a cannula was inserted. Lungs were instilled two times with 1 mL of sterile saline, and the bronchoalveolar lavage fluid (BALf) was recovered and pooled.

After plasma and BALf collection, the samples were decontaminated. For plasma, precipitation agent (acetonitrile + 2% acetic acid + 0.2% heptafluorbutyric acid) was added in a ratio of 1:2. Samples were vortexed, and incubated for 10 min at room temperature, followed by precipitation by centrifuging at 8500 rpm for 10 min at 20°C. Supernatant was stored at −80°C. For BALf, precipitation agent was added in a ratio of 1:2. Samples were incubated for 10 min at room temperature before storage at −80°C.

### Determination of polymyxin B1 and B2 levels

Polymyxin B concentrations in plasma and BALf were determined by a validated HPLC method at Pharmacelcus GmbH (Germany), with a lower limit of quantification (LOQ) of 0.0463 mg/L for polymyxin B2 in plasma and 0.0746 mg/L for plasma and BALf for polymyxin B1. For the analysis the HPLC system consisted of a Vanquish quaternary pump and a Vanquish split sampler (Thermo Fisher Scientific, USA). MS was performed on a Q-Exactive Plus mass spectrometer (Orbitrap™ technology with accurate mass) equipped with a heated electrospray interface (H-ESI) (Thermo Fisher Scientific, USA). The H-ESI was running in positive mode. Data were processed using the software Chromeleon version 7.2.10 ES MUf. The pump flow rate was set to 600 µL/min and the analytes were separated on a Synergy Polar RP Column, 2.5 μm 30 × 2.0 mm (Phenomenex, Germany). The HPLC was performed in the gradient mode using acetonitrile + 0.1% formic acid (A) as organic phase and water + 0.1% formic acid (B) as aqueous phase.

### Pharmacokinetic modelling

A population PK model was developed using non-linear mixed-effects modelling (NONMEM, version 7.4.4, ICON Development Solutions, Ellicott City, MD, USA). The analysis was performed using the FOCE method with INTERACTION on logarithmically transformed concentrations. Concentrations of polymyxin B were based on the sum of the B1 and B2 components. Below LOQ and zero values of summed concentrations were excluded from the analysis. Parameters were calculated for a virtual 1 kg mouse, resulting in PK parameters corresponding to a per kilogram base.

A structural model was developed, using one- and two-compartment models. The absorption following subcutaneous injections was described using an absorption rate constant (*k*_a_). Typical values for central (*V*_c_) and peripheral volume of distribution (*V*_p_), clearance (CL) and intercompartmental clearance (Q) were estimated. As bioavailability (F) could not be estimated, the PK parameter values corresponded to the ratios, such as CL/F and *V*/F. Addition of interindividual variability (IIV), described using an exponential model, was evaluated for each PK parameter. Residual variability was described using an additive error model for logarithmically transformed data. Minimum objective function values, parameter precision, error estimates and visual inspection of the goodness-of-fit plots were considered for model selection. The CIs around the final parameters were estimated by bootstrap with resampling (*n* = 500), and fit to the data was evaluated using visual predictive checks (VPCs) and VPCs stratified for dose (*n* = 500).

To calculate the unbound exposure in terms of *f*AUC_0–24h_/MIC an unbound fraction of 20% was used in the simulations, as reported earlier for polymyxin B in murine plasma.^4^

### Polymyxin B pharmacodynamics

Dose–response studies were performed for eight *A. baumannii* strains in both the thigh and lung infection models. Two hours after infection, treatment with polymyxin B [1–32 mg/kg every 12 h; total daily dose (TDD) 2–64 mg/kg] was administered subcutaneously over 24 h with three mice per dose level. Controls received the same volume of saline. Group size was based on results of previous studies.^3,4^ At start of treatment (*t* = 0), three mice were humanely killed to determine the bacterial load in each thigh or in lungs. All animals were humanely killed 24 h (*t* = 24 h) after the first dose, unless the welfare of the animals necessitated earlier termination, following animal welfare regulations. Thighs or lungs were aseptically removed, and homogenized in a gentleMACS M tube containing 2 mL PBS, using GentleMACS Dissociator (Miltenyi Biotec, Leiden, The Netherlands). A 10-fold dilution series was prepared and three drops of 10 µL were plated per dilution on MHA; these were incubated at 37°C for 16–18 h. Colonies were counted and the cfu per thigh or lung was calculated.

The drug effect was determined by the difference between the log_10_ cfu values at *t* = 0 (mean of three mice) and *t* = 24 h (value of individual mice) expressed as Δlog_10_ cfu. Using the *E*_max_ model with variable slope (GraphPad Prism version 9.5.1, USA), the *f*AUC/MIC values required for a static, 1-log_10_ and 2-log_10_ kill effect were determined.

### PTA in humans

The PTA for a dosing regimen of 200 mg every 24 h in hospitalized patients was determined using a previously published population PK model.^8^ The model used was based on therapeutic drug monitoring (TDM) samples taken from hospitalized patients treated for various types of infections.^8^ Monte Carlo simulation was used to estimate the *f*AUCs of 5000 patients taking into account a protein binding of 50% and 90% to represent the different values reported in literature (MicLab 2.71, Medimatics, The Netherlands).^9–12^ The PTA was calculated for isolates with polymyxin B MICs ranging from 0.25 to 8 mg/L.

## Results

### Polymyxin B pharmacokinetics

The PK profile of polymyxin B (B1 and B2 measured separately) in mouse plasma after single subcutaneous administration of 2–64 mg/kg is shown in Figure S1 (available as Supplementary data at *JAC* Online). The total peak plasma levels ranged from 1900 to 60 000 mg/L and 400 to 13 100 mg/L, for polymyxin B1 and polymyxin B2, respectively. The 64 mg/kg dose was not well tolerated, and mice in this group were terminated no later than after 2.25 h. Polymyxin B was not detectable in BALf.

### Pharmacokinetic modelling

A total of 136 polymyxin B1 concentrations (including 17 below LOQ) and polymyxin B2 concentrations (including 3 below LOQ) were available for the analysis. The PK profile was described by a one-compartment model with an additional absorption compartment. Inclusion of IIV on CL and *V* improved the model fit. The goodness-of-fit plots showed no large bias and the residuals were within the normal range (−2, 2). Relative standard error (RSE) values were within an acceptable range. Shrinkages remained high on CL and residual error. The estimates of the final model are presented in Table 1. The robustness of the model was tested via bootstrap (*n* = 500). A total of 295 successful runs were performed (37 were terminated during minimization and 168 due to boundary issues). The estimates of the bootstrap showed that the parameters were well estimated (Table 1). The VPC showed that the model provided an adequate fit overall and for most dosing regimens—except the highest, not tolerated, dose (64 mg/kg) (Figure S2).

**Table 1.** Parameter estimates of the final model and bootstrap

Parameter	Estimation	RSE, %	Shrinkage, %	95% CI	Bootstrap, median (2.5%–97.5%)
*k*_a_, h^−1^	7.57	35.3		2.33–12.80	7.59 (3.93–17.89)
*V*_c_, L/kg/F	1.64	7		1.42–1.87	1.62 (1.38–1.90)
CL, L/kg/h/F	0.27	9		0.23–0.32	0.27 (0.22–0.33)
IIV CL	32.6%	28.2	66		30.7% (10–49.9%)
IIV *V*	64.8%	57.7	19		61.4 (24.4–79.0%)
Additive error	0.27	47.1	51	0.021–0.52	0.26 (0.003–0.54)

CL, clearance; IIV, interindividual variability; *k*_a_, absorption coefficient; RSE, relative standard error; *V*_c_, central volume of distribution.

### Polymyxin B exposure response

**Figure 1.**
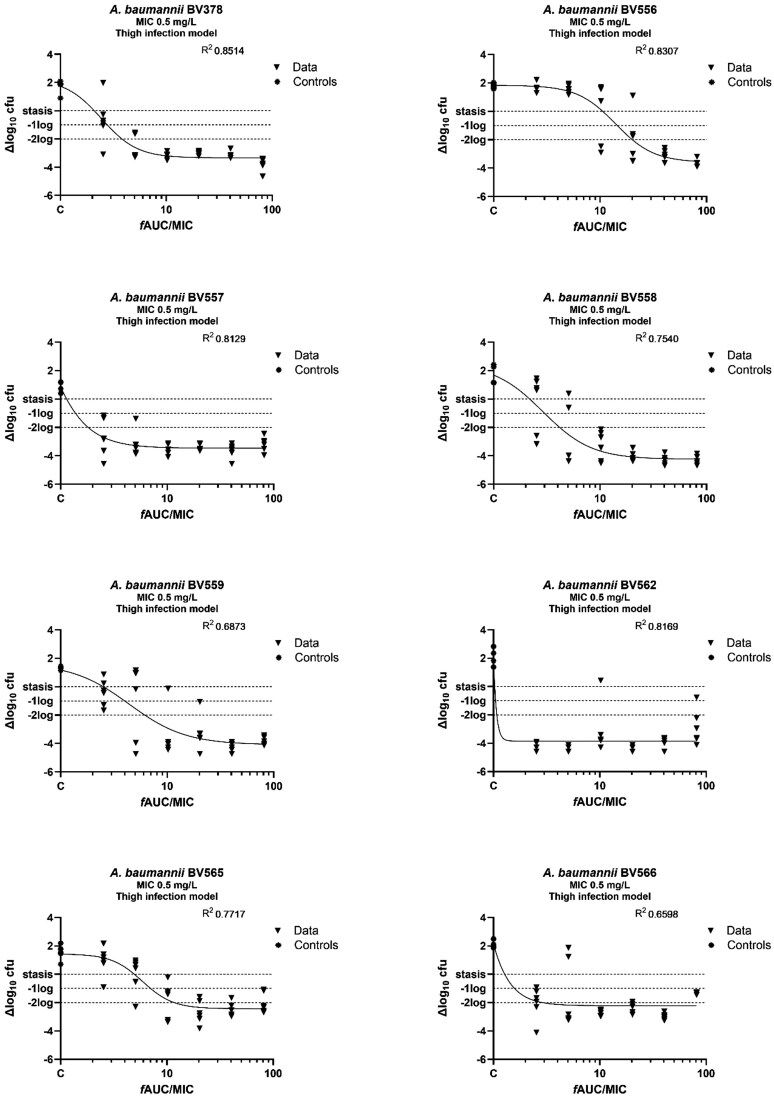
Exposure–response analysis of polymyxin B in the murine thigh infection model for eight *A. baumannii* strains. Neutropenic, thigh-infected mice received polymyxin B every 12 h. The drug effect (Δlog_10_ cfu) is the difference between log_10_ cfu values at *t* = 0 and *t* = 24 h. The controls represent mice not exposed to polymyxin B (set to 1).

**Figure 2.**
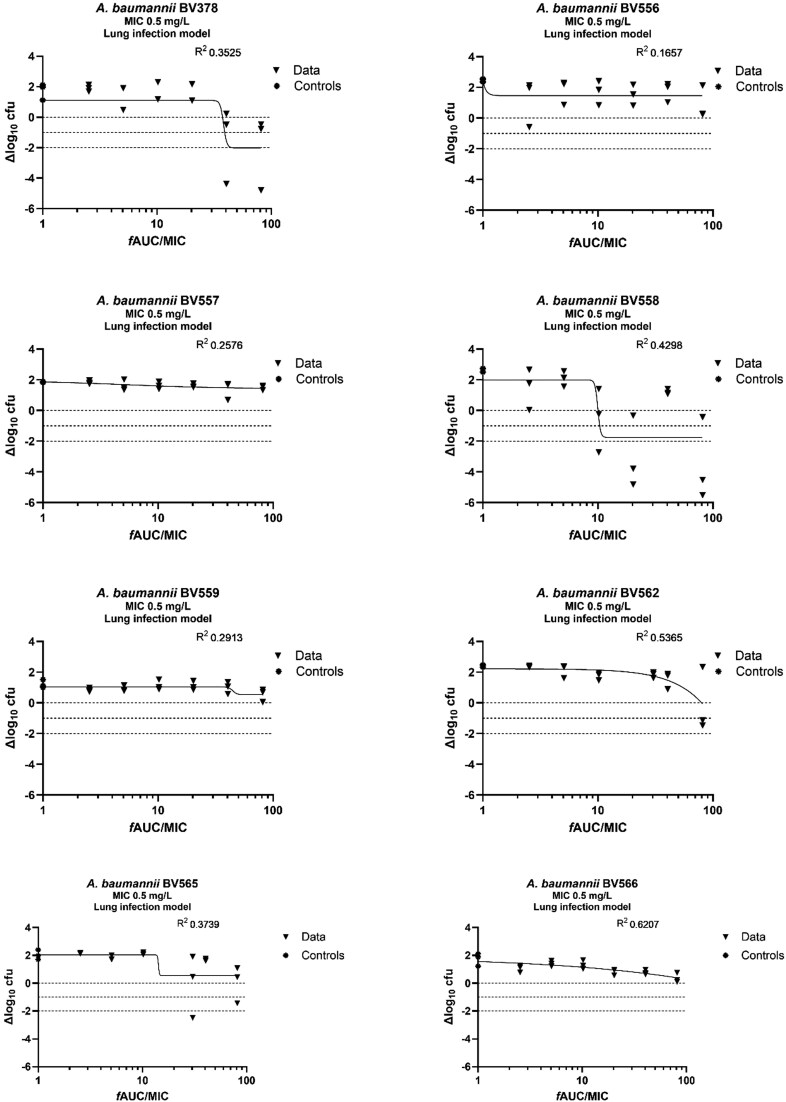
Exposure–response analysis of polymyxin B in the murine lung infection model for eight *A. baumannii* strains. Neutropenic, lung-infected mice received polymyxin B every 12 h. The drug effect (Δlog_10_ cfu) is the difference between log_10_ cfu values at *t* = 0 and *t* = 24 h. The controls represent mice not exposed to polymyxin B (set to 1).

The average bacterial burden at the start of the treatment (*t* = 0) was 1.9 × 10^6^ cfu/thigh (range: 6.5–72 × 10^5^ cfu/thigh) and 8.0 × 10^6^ cfu/lung (range: 1.8–22 × 10^6^ cfu/lung). Polymyxin B doses of 64 mg/kg were not tolerated. The exposure–response curves for the thigh model are shown in Figure 1. A static, 1-log_10_ kill and 2-log_10_ kill effects were found for all eight *A. baumannii* strains. In contrast, in the lung infection model (Figure 2), a 2-log_10_ kill effect was achieved in only 2/8 *A. baumannii* strains (BV378 and BV558). The exposure–response relationships of the eight strains were well described in the thigh infection model by the *E*_max_ model (*R*^2^ = 0.6598–0.8514). The *f*AUC/MIC values correlating with static, 1-log_10_ and 2-log_10_ kill effects for the individual strains in the thigh infection model are presented in Table 2. In contrast, in the lung infection model (Figure 2), a 2-log_10_ kill effect was achieved in only 2/8 *A. baumannii* strains (BV378 and BV558). The model fit was generally poor with low *R*^2^ values (0.1657–0.6207). The static, 1-log_10_ kill and 2-log_10_ kill targets were reached only in a minority of strains and therefore were unreliable (Table S1).

**Table 2.** Targets (*f*AUC/MIC) of polymyxin B in the murine thigh infection model

*A. baumannii* strain	Thigh infection model		
	Stasis	1-log_10_ kill	2-log_10_ kill
BV378	2.1	2.8	3.8
BV556	11	15	20
BV557	1.1	1.4	1.8
BV558	2.2	3.0	4.2
BV559	2.5	4.0	6.2
BV562	1.0	1.0	1.1
BV565	4.9	7.0	12
BV566	1.3	1.6	2.9
**Median**	2.2	2.9	4.0
**Mean**	3.3	4.5	6.5
**SD**	3.4	4.7	6.4

### PTA in humans

**Figure 3.**
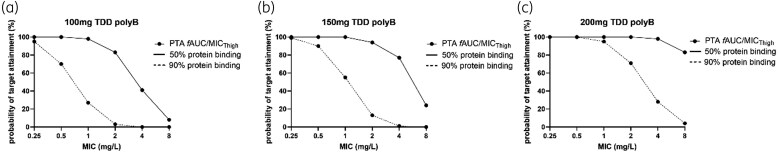
PTA in human plasma for the 100 mg (a), 150 mg (b) and 200 mg (c) TDD dosing regimen for the thigh model and 50% as well as 90% protein binding.

The PTAs for the 100, 150 and 200 mg clinical dose every 24 h (Figure 3) were performed taking into account a protein binding of 50% or 90%, as reported values for human protein binding in literature showed a wide range.^9–12^ In addition, the PK/PD target for the thigh infection model (median *f*AUC/MIC for 1-log_10_ kill of 2.9) was analysed. For a TDD of 100 mg and an MIC of 1 mg/L, or for a TDD of 200 mg and an MIC of 2 mg/L, the PTA using the PK/PD target derived from the thigh infection model was >95%, taking into account the low protein binding of 50%. The values for the PTA are shown in Table S2.

## Discussion

To determine efficacy of polymyxin B in *A. baumannii* infections, the PD of polymyxin B was studied in *A. baumannii* murine thigh and lung infection models using eight strains. In the thigh infection model, median *f*AUC/MIC values of 2.2, 2.9 and 4.0 were required to achieve stasis, 1-log_10_ kill and 2-log_10_ kill, respectively. In contrast, in the lung infection model there was insufficient killing to determine PD *f*AUC/MIC targets. Higher exposures in the murine infection model were not possible due to toxicity. For the PK/PD target derived from the thigh infection model and a protein binding of 90%,^8,11^ a PTA of >95% was reached only for strains with MICs up to 1 mg/L, indicating that current dosing regimens may be suboptimal in hospitalized patients.

Polymyxin B is a mixture of structurally related polypeptides, including the major components B1 and B2.^13^ The murine plasma PK of polymyxin B determined in the thigh infection model was based on these two major components, B1 and B2. As the lung infection model is more distressing for the animals, and we have shown previously that there is no difference in the PK between the two infection types for polymyxin B, we studied here PK only in the *A. baumannii* thigh-infected mice. Polymyxin B consists of more components than only B1 and B2; however, these two are the main components. Furthermore, methods used in clinical practice usually only include the B1 and B2 components.^14,15^ To be able to use the preclinically determined PK/PD targets in clinical practice, the method used to measure the polymyxin B concentrations should match those used to measure concentrations in human studies as well as those performed in clinical practice for TDM.

In the population PK analysis of our data, some saturation seemed to occur at higher dosages, probably in the absorption phase (16 mg/kg and up). As the 64 mg/kg doses were not tolerated, only concentrations up to 2 h after the start of treatment were available. This dose level could therefore not be adequately analysed. Excluding the concentrations of the 64 mg/kg group from the analysis did not affect *V*_c_ and CL estimates much: 1.64 versus 1.58 L/kg/F and 0.273 versus 0.265 L/kg/h/F. It did, however, affect the *k*_a_ and constant error, 0.27 versus 0.45, for, respectively, the model with and without the 64 mg/kg dosing regimen. The IIV decreased as well. We therefore accepted the final model with the 64 mg/kg concentration–time data. Landersdorfer *et al.*^3^ identified also a saturable absorption and a linear plus saturable elimination in the 16 and 32 mg/kg groups. In line with our analysis, the PK analysis of Jiao *et al.*^16^ did not improve after inclusion of a saturated clearance. Furthermore, both the high shrinkage and the low percentage of successful bootstraps run are caused because these data are scarce and taken from mice. It is possible to take only one sample per mouse, and the total number of animals used is limited for ethical reasons. This was taken into account in the inspection of both goodness-of-fit and VPC plots.

In the present study, a remarkable difference was found between the bacterial killing in the thigh and lung infection models. The PD targets associated with stasis, 1-log_10_ and 2-log_10_ kill in the murine thigh infection model were much lower compared with the limited killing observed in the murine lung infection model. This is in line with a previous study where PK/PD of colistin was explored in both the murine thigh and lung infection models. The response in the *A. baumannii* lung-infected mice was smaller as well.^17^ The structural similarity of polymyxin B and colistin may explain why this difference in infection models is observed for both compounds. This difference might be due to poor penetration of the polymyxins, as in our study polymyxin B concentrations in the BALf were not detectable. However, these findings contrast with the study of Jiao *et al.*^16^ in immunocompetent mice, in which BALf concentrations were detectable.

A limitation of the present study was that the MIC value from all *A. baumannii* isolates was 0.5 mg/L. As the current study is part of a project in which the efficacy of polymyxin B in combination with another compound is studied, the selection of strains was mainly based on MIC differences for the combination compound, rather than on differences in the polymyxin B MIC. In a study in 217 CRAB isolates, the polymyxin B MIC_50_ was 0.5 mg/L,^18^ and in a surveillance study the MIC_50_ of 2621 *Acinetobacter* spp. was found to be ≤1 mg/L.^19^ This shows that the MIC value of 0.5 mg/L is a frequently determined value for *A. baumannii*, and thus clinically relevant. Although the median MIC values for the strains were the same, there is considerable variability in the PD targets attributed to uncharacterized factors independent of the MIC.

The interpretation of the PD targets found in our study is limited by the limited breakpoints or distributions available. In the current breakpoint table of the EUCAST there are no breakpoints for polymyxin B. However, the United States Committee on Antimicrobial Susceptibility Testing (USCAST) has a susceptibility breakpoint for *A. baumannii* of S ≤2 mg/L. The breakpoints are not valid for respiratory tract infections or lower urinary tract infections, and it is recommended that the dosing regimen of 2.5 mg/kg/day should be combined with a second agent whenever possible.^20^ The USCAST recommendation that the breakpoint is not valid in respiratory tract infections is in line with the limited killing found in the murine lung infection model.

Although polymyxin B is currently being used as monotherapy in the treatment of *A. baumannii* infections, our study shows that its activity may be limited due to a low PTA, taking into account the USCAST breakpoint of 2 mg/L and a protein binding of 90%.^11,20^ The low values for the PTA were in line with the low PTA found previously in the murine thigh infection model for *E. coli* and *K. pneumoniae.*^4^ Since (nephro)toxicity of polymyxin B has been shown to be dependent on the plasma AUC, it might not be possible to increase dosing regimens.^21^ Values from 100 mg·h/L for the (total) AUC at steady state 24 h are associated with toxicity.^22,23^ Reported values for the protein binding in human plasma are variable, but values >90% are reported.^24^ Considering a protein binding of 90% in human plasma, an AUC of 100 mg·h/L translates to an *f*AUC of 10 mg·h/L. With MIC values of the circulating *A. baumannii* isolates of up to 2 mg/L, the *f*AUC/MIC values needed for efficacy will exceed the threshold values for toxicity.^25^ Using polymyxin B in combination with a second antibiotic may be a solution to preserve this drug for the future. One of the advantages of combination therapy is that the required exposure for a certain efficacy per drug component might be reduced compared with those needed in monotherapy. This may be an ideal way to avoid toxicity and increase efficacy.

In conclusion, the efficacy of polymyxin B monotherapy against *A. baumannii* was limited, especially in the murine lung infection model. Future studies are required to determine whether polymyxin B can be used in combination therapy with another compound, resulting in increased efficacy while avoiding toxicity.

## Supplementary Material

dkag097_Supplementary_Data

## Supplementary data

Figures S1 and S2 and Tables S1 and S2 are available as Supplementary data at *JAC* Online.

## References

[1] Velkov T, Roberts KD, Nation RL et al Pharmacology of polymyxins: new insights into an ‘old’ class of antibiotics. Future Microbiol 2013; 8: 711–24. 10.2217/fmb.13.39 23701329 PMC3852176

[2] Falagas ME, Kasiakou SK. Toxicity of polymyxins: a systematic review of the evidence from old and recent studies. Crit Care 2006; 10: R27. 10.1186/cc3995 16507149 PMC1550802

[3] Landersdorfer CB, Wang J, Wirth V et al Pharmacokinetics/pharmacodynamics of systemically administered polymyxin B against *Klebsiella pneumoniae* in mouse thigh and lung infection models. J Antimicrob Chemother 2018; 73: 462–8. 10.1093/jac/dkx409 29149294 PMC5890666

[4] van der Meijden A, Aranzana-Climent V, van der Spek H et al Pharmacokinetic and pharmacodynamic properties of polymyxin B in *Escherichia coli* and *Klebsiella pneumoniae* murine infection models. J Antimicrob Chemother 2023; 78: 832–9. 10.1093/jac/dkad022 36718051 PMC10377753

[5] Jiang X, Patil NA, Xu Y et al Structure-interaction relationship of polymyxins with lung surfactant. J Med Chem 2023; 66: 16109–19. 10.1021/acs.jmedchem.3c01497 38019899 PMC11608096

[6] Lacroix M, Moreau J, Zampaloni C et al *In vitro* pharmacokinetic/pharmacodynamic modeling of the effect of mucin on polymyxin B activity against *Acinetobacter baumannii*. Antimicrob Agents Chemother 2025; 69: e0153524. 10.1128/aac.01535-24 40135861 PMC12057341

[7] European Union . Directive 2010/63/EU of the European Parliament and of the Council of 22 September 2010 on the protection of animals used for scientific purposes. European Union, 2010.

[8] Kubin CJ, Nelson BC, Miglis C et al Population pharmacokinetics of intravenous polymyxin B from clinical samples. Antimicrob Agents Chemother 2018; 62: e01493-17. 10.1128/AAC.01493-17 PMC5826116 29311066

[9] Surovoy YA, Burkin MA, Galvidis IA et al Comparative polymyxin B pharmacokinetics in patients receiving extracorporeal membrane oxygenation. J Antimicrob Chemother 2022; 77: 1379–84. 10.1093/jac/dkac021 35134959

[10] Kwa AL, Lim TP, Low JG et al Pharmacokinetics of polymyxin B1 in patients with multidrug-resistant gram-negative bacterial infections. Diagn Microbiol Infect Dis 2008; 60: 163–7. 10.1016/j.diagmicrobio.2007.08.008 17916420

[11] Abodakpi H, Gohlke J, Chang KT et al Analytical and functional determination of polymyxin B protein binding in serum. Antimicrob Agents Chemother 2015; 59: 7121–3. 10.1128/AAC.01815-15 26324262 PMC4604353

[12] Sandri AM, Landersdorfer CB, Jacob J et al Population pharmacokinetics of intravenous polymyxin B in critically ill patients: implications for selection of dosage regimens. Clin Infect Dis 2013; 57: 524–31. 10.1093/cid/cit334 23697744

[13] Manchandani P, Dubrovskaya Y, Gao S et al Comparative pharmacokinetic profiling of different polymyxin B components. Antimicrob Agents Chemother 2016; 60: 6980–2. 10.1128/AAC.00702-16 27697755 PMC5075123

[14] Crass RL, Al Naimi T, Wen B et al Pharmacokinetics of polymyxin B in hospitalized adults with cystic fibrosis. Antimicrob Agents Chemother 2021; 65: e0079221. 10.1128/AAC.00792-21 34252297 PMC8448113

[15] Liu X, Yu Z, Wang Y et al Therapeutic drug monitoring of polymyxin B by LC-MS/MS in plasma and urine. Bioanalysis 2020; 12: 845–55. 10.4155/bio-2020-0051 32558589

[16] Jiao Y, Yan J, Vicchiarelli M et al Individual components of polymyxin B modeled via population pharmacokinetics to design humanized dosage regimens for a bloodstream and lung infection model in immune-competent mice. Antimicrob Agents Chemother 2023; 67: e0019723. 10.1128/aac.00197-23 37022153 PMC10190254

[17] Cheah SE, Wang J, Nguyen VT et al New pharmacokinetic/pharmacodynamic studies of systemically administered colistin against *Pseudomonas aeruginosa* and *Acinetobacter baumannii* in mouse thigh and lung infection models: smaller response in lung infection. J Antimicrob Chemother 2015; 70: 3291–7. 10.1093/jac/dkv267 26318190

[18] Thamlikitkul V, Tiengrim S, Seenama C. *In vitro* activity of polymyxin B against carbapenem-resistant *Acinetobacter baumannii*. J Med Assoc Thai 2014; 97: 1254–8. 25764631

[19] Gales AC, Jones RN, Sader HS. Global assessment of the antimicrobial activity of polymyxin B against 54 731 clinical isolates of gram-negative bacilli: report from the SENTRY antimicrobial surveillance programme (2001–2004). Clin Microbiol Infect 2006; 12: 315–21. 10.1111/j.1469-0691.2005.01351.x 16524407

[20] USCAST . Homepage. www.USCAST.org

[21] Zhao C, Mao W, Ye F et al Relationship between plasma polymyxin B concentrations and acute kidney injury in critically ill elderly patients: findings from a prospective study. J Int Med Res 2025; 53: 3000605251320733. 10.1177/03000605251320733 39956623 PMC11831629

[22] Wang P, Zhang Q, Zhu Z et al Comparing the population pharmacokinetics of and acute kidney injury due to polymyxin B in Chinese patients with or without renal insufficiency. Antimicrob Agents Chemother 2021; 65: e01900-20. 10.1128/AAC.01900-20 33168613 PMC7848972

[23] Han L, Xu FM, Zhang XS et al Trough polymyxin B plasma concentration is an independent risk factor for its nephrotoxicity. Br J Clin Pharmacol 2022; 88: 1202–10. 10.1111/bcp.15061 34449094

[24] Deng Y, Gu JY, Li X et al Does monitoring total and free polymyxin B1 plasma concentrations predict polymyxin B-induced nephrotoxicity? A retrospective study in critically ill patients. Infect Dis Ther 2022; 11: 1591–608. 10.1007/s40121-022-00655-3 35689791 PMC9334479

[25] Zha L, Zhang X, Cheng Y et al Intravenous polymyxin B as adjunctive therapy to high-dose tigecycline for the treatment of nosocomial pneumonia due to carbapenem-resistant *Acinetobacter baumannii* and *Klebsiella pneumoniae*: a propensity score-matched cohort study. Antibiotics (Basel) 2023; 12: 273. 10.3390/antibiotics12020273 36830183 PMC9952519

