# Supplementary material for: The pharmacodynamics of polymyxin B in Acinetobacter baumannii in murine thigh and lung infection models
Source: J Antimicrob Chemother. 2026 Mar 17;81(4):dkag097. doi: 10.1093/jac/dkag097 (PMC13017451; doi:10.1093/jac/dkag097)
Supplement: dkag097_Supplementary_Data [file dkag097_supplementary_data.docx]

**Supplementary data**

**Figure S1: concentration-time profiles of polymyxin B1 and B2**


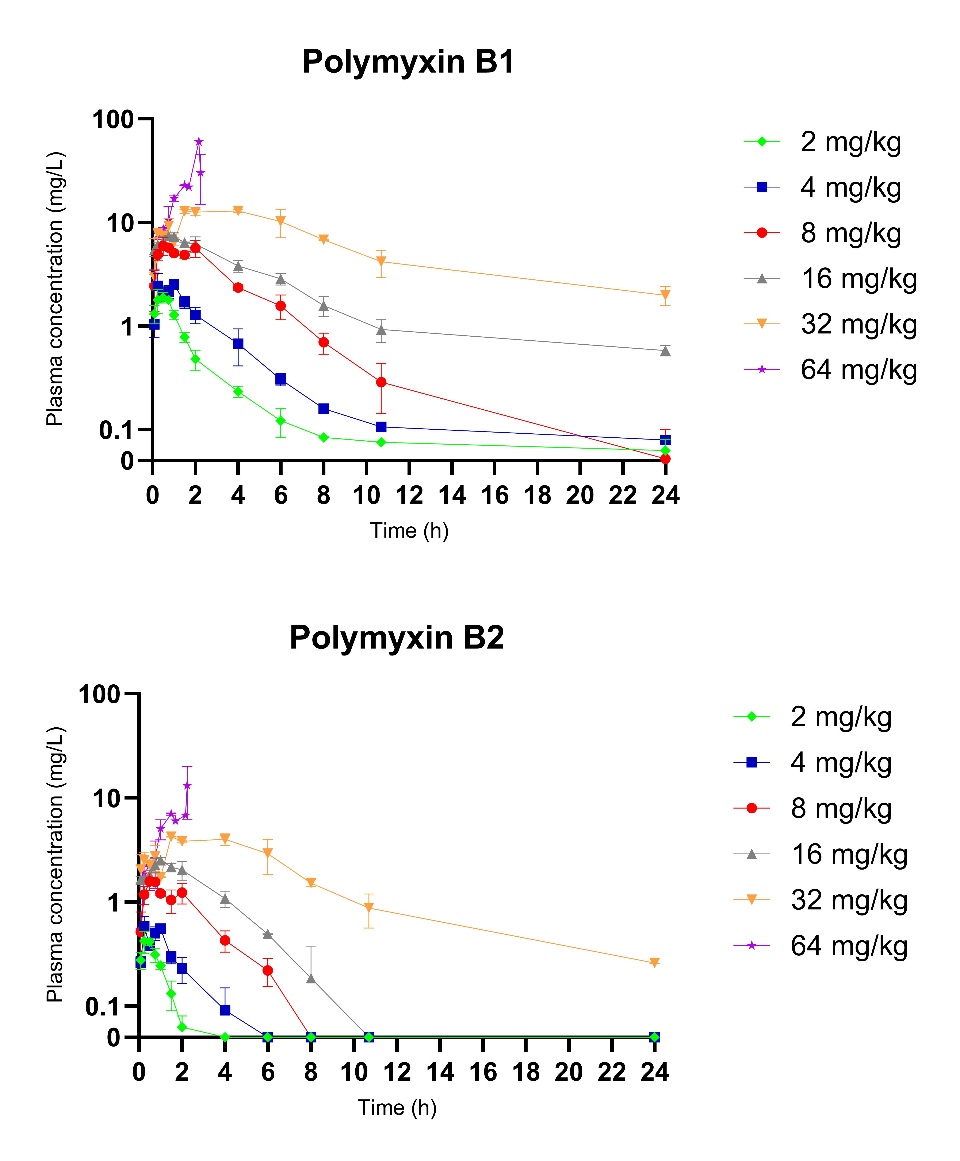


Concentration-time profiles of polymyxin B1 and B2 in murine plasma for the different doses (2-64 mg/kg). The lower limit of quantification (LOQ) was 0.0746 mg/L for plasma for polymyxin B1 and 0.0463 mg/L for polymyxin B2 in plasma.

Figure S2: Visual predictive checks of the final model.


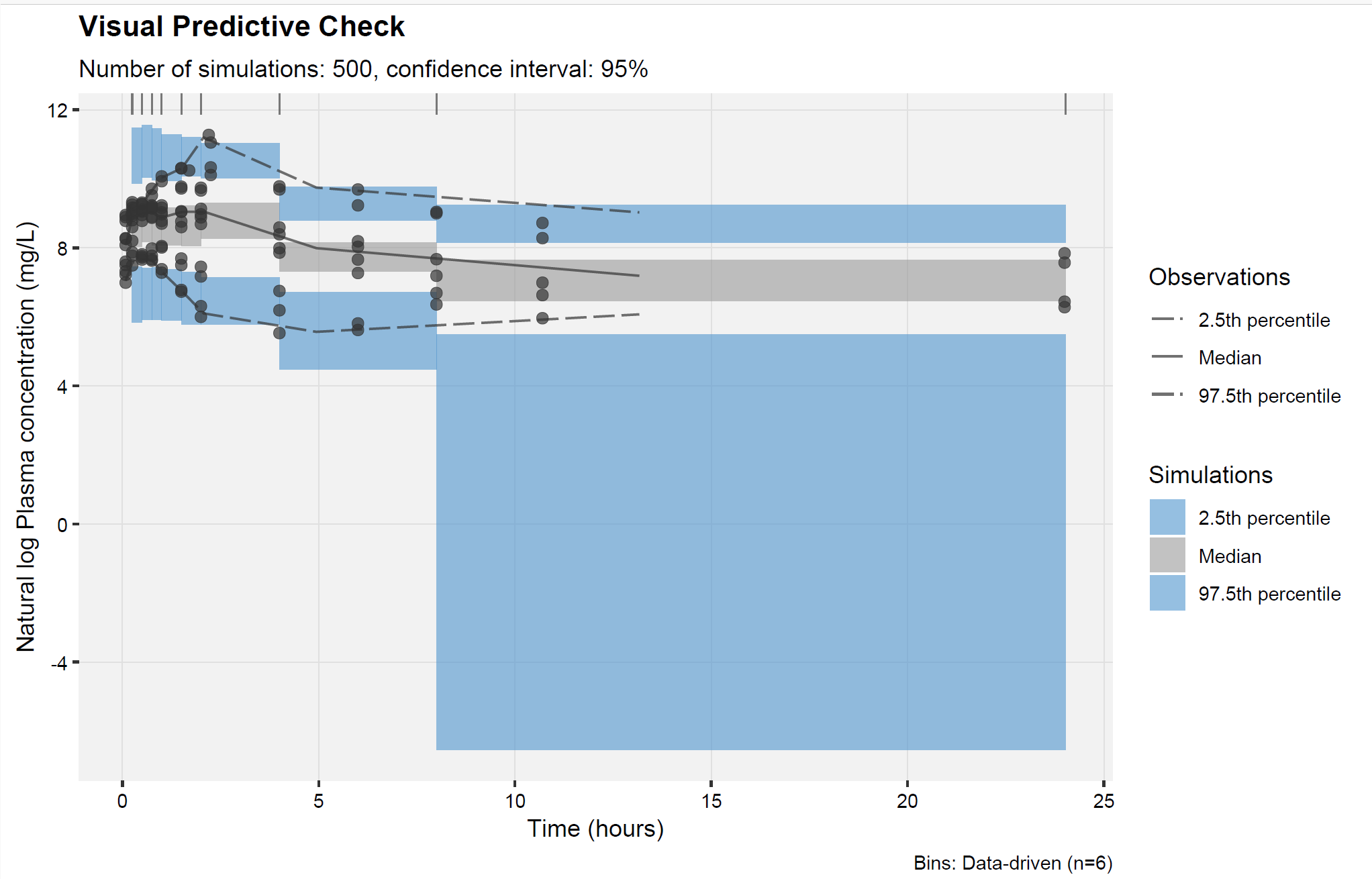


Visual predictive checks of the final model all dosing regimen combined. Dots and lines represent observed data (solid line: median, dashed lines: 95% CI) and shaded areas the model predicted medians (grey) and 95% CIs (blue).

Table S1: Targets (*f*AUC/MIC) of polymyxin B in the murine lung infection model.

| *A. baumannii* strain | **Lung infection model** | | |
| --- | --- | --- | --- |
|  | Stasis | 1-log_10_ kill | 2-log_10_ kill |
| BV378 | 38 | 39 | 45 |
| BV556 | NA | NA | NA |
| BV557 | NA | NA | NA |
| BV558 | 10 | 10 | NA |
| BV559 | NA | NA | NA |
| BV562 | 80 | 101 | 120 |
| BV565 | NA | NA | NA |
| BV566 | 164 | 598 | NA |

NA: Not achieved

Table S2: Table of the PTA for the 3 dosing regimens for the thigh PD-target (PTA *f*AUC/MIC_Thigh_)

|  | **200 mg TDD** | | **150 mg TDD** | | **100 mg TDD** | |
| --- | --- | --- | --- | --- | --- | --- |
| **MIC (mg/L)** | **50% PB** | **90%PB** | **50% PB** | **90%PB** | **50% PB** | **90%PB** |
| **0.25** | 100 | 100 | 100 | 99 | 100 | 95 |
| **0.5** | 100 | 100 | 100 | 90 | 100 | 70 |
| **1** | 100 | 95 | 100 | 55 | 98 | 27 |
| **2** | 100 | 71 | 94 | 13 | 83 | 3 |
| **4** | 98 | 28 | 77 | 1 | 41 | 0 |
| **8** | 83 | 4 | 24 | 0 | 8 | 0 |
